# Towards better understanding of factors contributing to medical physicist well‐being in academic medical centers: A systems‐analysis approach

**DOI:** 10.1002/acm2.70122

**Published:** 2025-05-11

**Authors:** Elizabeth Kwong, Chao Chin Liu, Karthik Adapa, Lisa Vizer, Brian Anderson, Damian McHugh, Todd Pawlicki, Moyed Miften, Amit Sawant, Nadia Charguia, Shiva Das, Lawrence B. Marks, Jean L. Wright, Lukasz Mazur

**Affiliations:** ^1^ Carolina Health Informatics Program, Division of Healthcare Engineering University of North Carolina at Chapel Hill School of Information and Library Science, University of North Carolina at Chapel Hill School of Medicine Chapel Hill North Carolina USA; ^2^ Division of Healthcare Engineering University of North Carolina at Chapel Hill School of Medicine Chapel Hill North Carolina USA; ^3^ Department of Computer Science Georgetown University Washington, DC USA; ^4^ Department of Radiation Oncology University of North Carolina at Chapel Hill School of Medicine Chapel Hill North Carolina USA; ^5^ Curi Raleigh North Carolina USA; ^6^ Department of Radiation Medicine University of California San Diego School of Medicine La Jolla California USA; ^7^ Department of Radiation Oncology University of Colorado School of Medicine Aurora Colorado USA; ^8^ Department of Radiation Oncology University of Maryland School of Medicine Baltimore Maryland USA; ^9^ Integrated Well‐being Program University of North Carolina at Chapel Hill School of Medicine Chapel Hill, North Casrolina USA

**Keywords:** burnout, medical physicists, radiation oncology, NAM model, systems analysis, well‐being

## Abstract

The well‐being of medical physicists can impact overall system performance, patient safety, and quality of patient care. There are limited formal assessments of factors contributing to physicists well‐being. Nine medical physicists at a US academic medical center were surveyed on 21 workplace factors, drawn from the National Academy of Medicine's systems model of clinician burnout and professional well‐being between May 2022 and August 2022. Highly rated factors were summarized and presented to medical physicists in focus groups. Contextual inquiries (a form of shadowing) were conducted to gather additional information about factors contributing to well‐being. Qualitative data from the survey, focus groups, and contextual inquiries were used to generate an affinity model, which medical physicists then validated and used to prioritize top factors. Twenty‐two medical physicists at the academic medical center and three other US academic medical centers rated these factors by level of impact and level of effort, and improvement recommendations were made based on these results. Key factors affecting medical physicist well‐being included inadequate staffing, work‐life integration, excessive workload, and time pressure. Twenty‐two medical physicists across four institutions prioritized the following top factors for improvement: (i) retain the hybrid work model, (ii) hire additional medical physicists to cover clinic responsibilities, (iii) limit or compensate after hours work, (iv) improve scheduling workflows, and (v) improve communication and visibility from organization‐level leadership and administration. High impact, low effort priorities to improve medical physicist well‐being across the four institutions include work‐life integration, scheduling workflows, and relationships with leadership. These factors seem to be within the improvement control of each radiation oncology center. Further research is needed to establish the generalizability of our findings and spearhead broad policy changes.

## 1 INTRODUCTION

Among radiation oncology professionals, medical physicists have relatively high workloads and experience challenging work environments, which can affect well‐being.^1^ Suboptimal levels of well‐being are associated with increased job‐withdrawal, poorer quality of patient care, and a higher incidence of medical errors that impact patient safety and can lead to patient harm.^2^, ^3^ Consequently, the identification of factors associated with improved well‐being and lower burnout among medical physicists are essential with regard to patient safety and quality of patient care.

Although several international studies quantifying the levels of burnout in radiation oncology settings exist, few studies assess the *factors* contributing to well‐being among medical physicists. Of these, factors related to well‐being have predominantly been assessed using only surveys and questionnaires.^4^, ^5^, ^6^, ^7^ These studies acknowledge that assessment solely via survey has limitations, including potential response bias.^4^, ^7^ Consequently, studies using other approaches, analyses, and data collection methods are needed to holistically capture factors contributing to burnout. While burnout and well‐being are often not explicitly mentioned in quality and safety reports, this report supports the use of systematic analysis of well‐being to improve safety and associated quality management opportunities in radiation oncology settings.^8^

A systems‐analysis approach is suited to evaluating well‐being because it incorporates knowledge of stakeholders and their goals, activities, technologies, and the environments they operate within to understand the system and its complexities as a whole.^9^ This approach has been applied within several diverse areas, including obesity prevention,^10^ mental health treatment,^11^ and the COVID‐19 pandemic,^12^ but there have been few studies using a systems‐analysis approach to address well‐being. The National Academy of Medicine's systems model of clinician burnout and professional well‐being (NAM model) uses a systems‐analysis approach to outline work system factors that contribute to clinician burnout and well‐being. To the best of our knowledge, previous studies have not analyzed factors associated with medical physicist well‐being using a systems‐analysis approach and associated data collection methods. The purpose of this work is to use a systems‐analysis approach to study well‐being and associated factors among medical physicists in radiation oncology and assess its magnitude. By assessing contributing factors using the NAM model, health systems may be better equipped to improve medical physicist well‐being.^9^

## 2 METHODS

### 2.1 Study overview

**FIGURE 1:**
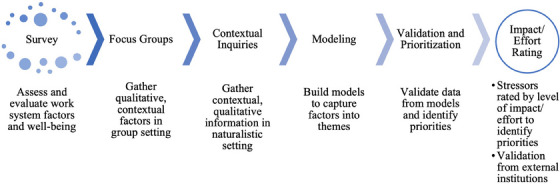
Study overview.

A sequential, mixed‐methods, participatory, and data‐driven study design based on the contextual design approach^13^ was used to gather, analyze, and model well‐being data among radiation oncology medical physicists at a single US academic medical institution. Recruitment and all steps of the research (Figure 1) occurred between May and August 2022. The study protocol was reviewed and approved by the Institutional Review Board. The study included a survey, focus groups, contextual inquiries, modeling, a validation and prioritization session, and impact/effort rating summarized in Figure 1.

The study started with a survey as the broad data collection method for qualitative and quantitative data on well‐being and workplace factors. Subsequently, focus groups were guided by survey results, with survey data presented back to medical physicists so that they could provide additional contextual information for top‐rated factors. Focus groups were followed by contextual inquiries to gather further qualitative information in a naturalistic setting. The data from these three data collection methods were used to build an affinity model (diagram/tool to organize ideas into themes) to comprehensively capture data and sort them into themes. The order of our study allowed the research team to start with broader data gathering methods and gradually move towards more narrow, specific, and detailed data collection.

### 2.2 Participating institutions

Medical physicists from four US academic medical institutions located in the US West, Southeast, and Mid‐Atlantic participated in this study (Table 1). Surveys, focus groups, contextual inquiries, modeling, and validation and prioritization were conducted at Institution A to “drill‐down” on the possible factors related to well‐being at one institution. Impact/Effort rating was conducted at Institutions A, B, C, and D to ensure priorities were applicable to a broader medical physicist population. Results from the Impact/Effort rating were presented in aggregate among all four institutions per IRB directive.

**TABLE 1:** Participating institutions and relevant information.

	Institution A^a^	Institution B	Institution C	Institution D
**Facility Size**	3345 sq m	2787 sq m	3345 sq m	19416 sq m
**Number of machines**	6	4	9	16
**Number of radiation therapy patients treated (per year)**	2500+	3000+	3000+	3500+
**Number of staff/ faculty (Total)**	54	76	84	>70
**Medical physicists**	11	19	14	28
**Physicians**	12	21	12	22
**Dosimetrists**	7	14	12	25
**Therapists**	19	22	31	57
**Other (e.g., APPs, residents)**	5		15	

^a)^Participated in initial survey, focus groups, contextual inquiries, modeling, and validation and visioning session in addition to impact/effort rating.

### 2.3 Data collection and analysis

#### 2.3.1 Survey

A survey was administered by email using a survey communications platform (Qualtrics) to all medical physicists in the radiation oncology department at Institution A (Supplementary Material, Figure A). The survey included 21 general workplace factors based on the NAM model.^9^ Participants provided their perceptions regarding (i) the severity of factors (degree to which the factor contributed to their well‐being) on a 5‐item Likert scale, and (ii) the priorities for improvement (priority the institution should take in addressing and improving this factor) on a 4‐item Likert scale. Participants could also clarify anything pertaining to that factor via open‐ended response section listed next to each factor and could also write in their own contributing factors if they felt like the 21 general workplace factors did not capture something in particular. Of the 21 factors in the NAM model, 10 are categorized as “job demands”—various intrinsic aspects of clinical work and work inefficiencies—and 11 are categorized as “job resources”—the tangible and intangible resources within the work environment (see Table 2 under results for the 21 stressors and their respective categorizations). Refer to Supplementary Materials, Figure 1 for the electronic survey.

**TABLE 2:** Ratings and ranking of the extent to which workplace factors contribute to medical physicist well‐being (Institution A).

	Severity		Priority	
Workplace Stressor	Rank	Mean (SD)	Rank	Mean (SD)
Inadequate staffing^a^	1	3.13 (1.25)	1	2.50 (0.55)
Work‐life integration^b^	1	3.13 (1.25)	4	1.83 (0.75)
Excessive workload^a^	3	2.88 (1.13)	2	2.16 (0.41)
Time pressure^a^	4	2.63 (1.19)	5	1.66 (0.82)
Lack of dedicated time for professional development requirement^b^	5	2.50 (0.93)	11	1.29 (0.49)
Inefficient workflows^a^	5	2.50 (1.07)	8	1.50 (1.00)
Lack of support for research and teaching^b^	5	2.50 (1.20)	5	1.67 (0.52)
Unmanageable Work Schedules^a^	8	2.38 (1.19)	3	1.86 (0.69)
Administrative Burden^a^	9	2.25 (0.71)	13	1.00 (0)
Interruptions & Distractions^a^	10	2.13 (1.13)	13	1.00 (0)
Intrinsic Motivations and Rewards^b^	10	2.13 (1.13)	13	1.00 (0)
Extrinsic Motivations and Rewards^b^	12	2.00 (0.76)	5	1.67 (0.82)
Values and Expectations Alignment^b^	13	1.88 (0.99)	10	1.33 (0.58)
Lack of Recognition for Quality Improvement Activities^b^	13	1.88 (1.13)	13	1.00 (0)
Job Control (Flexibility and Autonomy)^b^	13	1.88 (1.13)	8	1.50 (0.58)
Inadequate Technology Implementation^a^	16	1.75 (0.46)	13	1.00 (0)
Professional Relationships^b^	17	1.63 (0.74)	12	1.20 (0.45)
Organizational Culture^b^	17	1.63 (0.74)	13	1.00 (0)
Physical Work Environment^b^	19	1.50 (0.53)	13	1.00 (0)
Patient Stressors^a^	20	1.38 (0.52)	13	1.00 (0)
Moral Distress^a^	21	1.25 (0.46)	13	1.00 (0)

^a)^ Job demands.

^b)^ Job resources.

#### 2.3.2 Focus groups

To accommodate medical physicists’ schedules, three focus groups were conducted virtually using a focus group guide (Supplementary Materials, Figure B) and a video communications platform to accommodate hybrid work schedules at Institution A. Focus groups were facilitated by experienced research team members. The purpose of these groups was to gather additional information on top‐ranked workplace factors from the survey. The quantitative results for the top‐rated work system factors and top‐ranked priorities, plus the associated textual survey responses, were summarized and presented to each focus group. Participants were given time to read and reflect on each factor. They were asked open‐ended questions to promote discussion and to elicit more contextual information about each of the factors. Participants were asked to focus on describing the problems in the current system and providing detailed contextual information, rather than speculating about possible solutions. At the end of each focus group, participants were asked to prioritize the key factors to identify how and if any factors had shifted in priority for medical physicists.

#### 2.3.3 Contextual inquiry

Contextual Inquiries are observations conducted in naturalistic settings (everyday workplace, e.g., office, clinic) to better understand human behavior in that context.^13^ For this study, contextual inquiries were used to gather information about key factors contributing to well‐being among radiation oncology medical physicists at Institution A and gain insight into systems and day‐to‐day operations. Participants had the option to end the contextual inquiry at any point if they so desired. Contextual inquiries involved one or two experienced research team members trained in contextual design shadowing one participant, allowing them to describe their tasks when appropriate, and asking questions when it was not intrusive. Each shadowing session was followed by a 30‐min semi‐structured interview to provide participants an opportunity to further elaborate on key factors (Supplementary Materials, Figure C). Interviews were conducted in private locations (e.g., conference room, empty break room). Observations and responses were captured by research team members via notetaking. At the end of each contextual inquiry, the research team member reviewed the notes with the participant to ensure all information was captured accurately and that anything the participant did not want shared was removed from the notes. Each contextual inquiry was presented at a weekly interpretation session with the research team to report and discuss the key findings from contextual inquiry sessions.

#### 2.3.4 Affinity model

An affinity model is a diagram or tool used to organize ideas or data into relationships or themes.^14^ Using qualitative data from the survey, focus groups, and contextual inquiries, an affinity model was built to aggregate, identify, and categorize the key themes and factors using the NAM model and methodology described by Holtzblatt and Beyer.^9^, ^13^ The affinity model was created by research team discussion of data points, taking each individual factor as a data point and organizing the data points into themes and sub‐themes based on team consensus of their perceived similarity. By grouping data points into thematic categories, an overarching structure emerges which demonstrates how factors interconnect.

#### 2.3.5 Validation and prioritization

The model was then presented to medical physicists at Institution A to validate the data by leaving the text black if they agreed with a data point, marking the text in red if they disagreed with a data point, and amending or annotating any data points in blue text. Medical physicists were also asked to prioritize their top five factors contributing to well‐being for improvement.

#### 2.3.6 Impact/effort rating across institutions

The priorities from the validation and prioritization session were compiled by a research team member into a brief survey (Supplementary Materials, Figure D), and medical physicists at all four institutions (Institutions A, B, C, and D) classified the sorted priorities by level of impact (high, medium, and low impact) and level of effort (high, medium, and low effort). The level of impact was defined by how much value or impact the outcomes will have on the division. The level of effort was estimated based on the perceived time, money, resources, and capacity that would be needed to achieve the desired outcome. The survey and summary of the study purpose were distributed via electronic mail by division leads and research team members, and medical physicists were asked to complete the voluntary survey in 2–3 weeks. Results were reported in aggregate among the four institutions.

## 3 RESULTS

Eleven medical physicists at Institution A were offered the opportunity to participate in the study. Two declined at the beginning of the study; the results of the nine (9/11 = 81.8%) participants are reported. Years worked in their current positions ranged from <1 year to >20 years (median: 3–9 years), hours worked ranged from 40–59 h (median: 40–49 h), and age groups ranged from 25–54 (median: 35–44 years). Because of the small number of possible participants, we do not disclose or break down data by demographics to preserve the privacy of participants.

### 3.1 Survey results

Eight of the nine medical physicists responded to the electronic survey between May 19 2022 and June 2 2022. One medical physicist did not complete the survey. The key factors affecting medical physicist well‐being were perception of inadequate staffing (mean [SD] 3.1 [1.2]), work‐life integration (3.1 [1.2]), excessive workload (2.8 [1.1]), and time pressure (2.6 [1.2]). Table 2 provides a full list of factors, including severity and priority ranks.

### 3.2 Focus groups

Six of the nine medical physicists attended the focus groups, each facilitated by two research team members, on June 8–9 2022. Participants provided in‐depth contextual information about the key factors affecting their well‐being.

At the end of each focus group, participants were asked to prioritize the key factors. Prioritization results indicate that inadequate staffing remained the top priority for medical physicists. Work‐life integration was moved to a higher priority (2nd priority rank vs. 4th before focus groups) in addition to extrinsic motivation and rewards (3rd priority rank vs. 5th before focus groups).

### 3.3 Contextual inquiries

78% (*n* = 7 of 9) of medical physicists participated in contextual inquiries between June 2022 and July 2022. Research team members observed seven medical physicists for a total of 27.25 h of contextual inquiries. Qualitative data were gathered from observations, comments, and interview responses among the 7 medical physicists, with several common themes and sub‐themes that were consolidated on the affinity model and are presented in the section below.

### 3.4 Affinity model

Qualitative data gathered from the contextual inquiries, semi‐structured interviews, focus group discussions, and survey comments were compiled to develop the affinity model. This model summarizes for medical physicists the key factors for them to scrutinize during the validation and prioritization sessions. Contextual information from survey comments, focus groups, and contextual inquiries resulted in 128 possible factors that were captured and organized on the affinity model. The number of factors for main‐ and sub‐themes is presented in Table 3. The “General” category under sub‐themes captured any general or “other” data points related to the key factor that did not fit into one of the other listed sub‐themes.

**TABLE 3:** Key factors and sub‐themes within each factor captured in the affinity model.

Factors (#)	Sub‐themes (#) within each factor	Percentage of medical physicists in agreement after validation
Inefficient workflows^a^ (28)	General (7) Uncertainty (and unpredictability in clinical load) (6) Meeting inefficiencies (6) Scheduling (5) Policies and procedures (2) Other hospitals (with different workflows) (2)	96%
Inadequate technology implementation^a^ (24)	Machine issues (6) Machine repairs (5) Engineers/vendors (4) Treatment delivery hardware (3) Treatment delivery software (2) Treatment planning system (1) General (2) Information Service Division (1)	94%
Lack of protected /dedicated time^b^ (13)	General (6) Development days (5) Protected time for educational activities (2)	97%
Work‐life integration^b^ (9)	General (6) Hybrid work (3)	100%
Training (9)	Training (9)	97%
Extrinsic motivation^b^ (9)	Extrinsic motivation (9)	100%
Excessive workload^a^ (8)	General (6) Chart check (2)	91%
Inadequate staffing^a^ (7)	Inadequate staffing (7)	98%
Organizational culture^b^ (7)	System leadership (4) System administration (3)	100%
Lack of support for research and teaching^b^ (7)	Teaching (4) Research (3)	96%
Interruptions & distractions^a^ (4)	Interruptions & distractions (4)	96%
Time pressure^a^ (3)	Time pressure (3)	100%
**Average overall agreement across all themes and sub‐themes**		**96%**

^a^ Job demands.

^b^ Job resources.

### 3.5 Validation and prioritization

Validation and prioritization of key factors were performed by 78% (*n* = 7) of medical physicists at Institution A between July 2022 and August 2022. There was consensus (100% agreement among medical physicists) for 76.6% of the factors depicted in the affinity model. 23.4% (*n* = 30) of the breakdowns had some level of disagreement (1 or more medical physicists disagreed with the factor). See Table 3 (last column) for detailed information.

**FIGURE 2:**
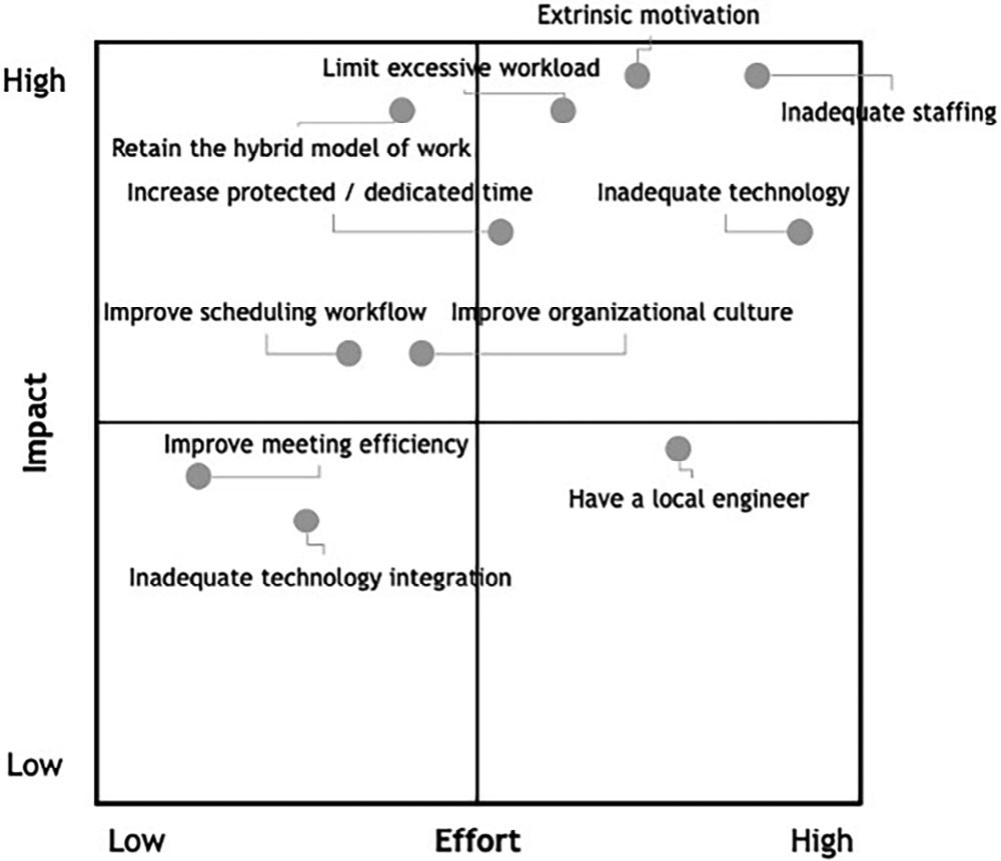
Level of effort versus level of impact matrix based on prioritizations from medical physicists across four US academic medical institutions (*n* = 22).

Medical physicists were also provided the opportunity to amend, annotate, or add any statements on the affinity model when validating the data. Thirteen amendments/annotations and six additional statements were included in the compiled, validated affinity model. Amendments and annotations included comments that some of the factors mentioned were part of the role or being in healthcare (e.g., adjusting schedule to address a machine issue), an emphasis on the need for additional staffing and compensation for covering tasks when understaffed, and commentary on how scheduling might be improved. After each of the seven medical physicists prioritized their top five factors from the affinity model, duplicates were removed and common themes were combined to form a total of eleven priorities for improvement (Figure 2 and Table 4).

**TABLE 4:** Medical physicist‐generated summary of top priorities to improve well‐being.

Impact/effort rating	Priority	Description
High impact, low effort	Retain the hybrid model of work	Maintaining a hybrid model of work would be beneficial moving forward. It is important to physicists’ work‐life balance and well‐being.
	Improve scheduling workflow^a^	Scheduling workflows not scheduled out several weeks in advance can lead to unpredictability and difficulty in planning. It would be beneficial having the schedule out one month in advance and in a timelier manner so that medical physicists can appropriately plan activities, vacation time, etc.
	Improve organizational culture^b^	Communication and visibility from hospital (not department) leadership and administration should be improved. The hospital can show they value and care about their employees through leadership visibility (interfacing with employees), listening and acting on feedback, and pay/benefits.
High impact, high effort	Extrinsic motivation^b^	Improved pay and benefits as well as bonuses for working overtime would help with employee retainment.
	Inadequate staffing^a^	Having more medical physicists to cover clinical physics responsibilities would help ease workload demands.
	Limit excessive workload^a^	Excessive workload extending into evenings and post‐work hours disrupts work‐life balance, often driven by tight treatment schedules. Compensation is often not provided for this extra work.
	Increase protected/dedicated time^b^	Protected and/or dedicated time is needed for educational responsibilities (i.e., staff training, mentoring, professional development) and updating policy and procedures. Allocation of time for these tasks would reduce pressure to attend to these tasks while managing clinical responsibilities.
	Inadequate technology^a^	Machines, dosimetry systems, and QA devices should be upgraded in a timely manner. They should be more reliable; when they break down, service to fix them is suboptimal.
Low impact, low effort	Inadequate technology integration^a^	People log machine issues but don't always follow up with engineers to get the issues fixed. If machine issues are left unattended, they can potentially become bigger extrinsic issues.
	Improve meeting efficiency	Meetings should be more efficient (e.g., meaningful use of time).
Low impact, high effort	Have a local/on‐site/dedicated engineer	Having a local, on‐site, or dedicated engineer to address machine breakdowns or issues would be beneficial. This would allow machine issues to be fixed more quickly, improving patient care timelines and minimizing long wait times and late evenings spent by medical physicists.

^a^ Job demands.

^b^ Job resources.

### 3.6 Impact/effort rating

Medical physicists from all four institutions (*n* = 22; Institutions A [*n* = 4], B [*n* = 1], C [*n* = 11], and D [*n* = 4], did not disclose association with institution [*n* = 2]) rated the 11 priorities by perceived level of impact and level of effort respective to their institution in August 2022 (Figure 2). A summary description of priorities to improve medical physicists well‐being is presented in Table 4.

## 4 DISCUSSION

This study used a systems‐analysis approach to evaluate and prioritize factors impacting well‐being among radiation oncology medical physicists at US academic medical centers. Using multimodal data collection methods resulted in a comprehensive understanding of medical physicists and underlying factors impacting well‐being that may not have been uncovered through one method. This study differs from previous studies assessing factors contributing to burnout among radiation oncology professionals (including medical physicists) using just surveys.^4^, ^5^, ^6^, ^7^ Our study went beyond identifying the need for medical physicist support; the study assessed factors holistically and identified specific priorities for targeted improvement by medical physicists themselves.

Overall, the prioritized factors were similar to key factors identified in other studies within radiation oncology settings (e.g., inadequate clinical and administrative support, imbalanced personal and professional lives, increased time in electronic medical records, and lack of transparency from hospital leadership and inclusion in administrative decisions).^15^ Other studies outside radiation oncology settings similarly cite excessive workloads, workplace inefficiencies, work‐life integration, and technology as key factors among physicians and other healthcare professionals.^1^, ^16^, ^17^ However, several factors were unique to medical physicists. For example, a hybrid work model was cited as key to work‐life integration and medical physicist well‐being; this is not often noted by other healthcare professionals since their roles are not conducive to a “work‐from‐home” mode. Additionally, a lack of protected/dedicated time for professional and educational development requirements was not typically noted as a key factor in other studies among other health professionals,^15^, ^17^ but this may simply be reflective of our study being conducted at centers with a strong academic focus.

Overall, our results suggest that the majority of factors perceived as the highest severity and priority are job *demands* rather than job *resources* (see Table 3; as categorized by the NAM model).^9^ The results from our work are similar to studies that have used job demands and job resources models to evaluate the impact of work systems on healthcare professionals’ burnout.^18^, ^19^, ^20^ While these studies recognized the significance of both decreasing job demands and improving job resources, one study acknowledged that a far greater impact would result from addressing job demands that increase perceived effort.^19^ Importantly, our study and others note the interconnectedness between several of the factors across job demands and job resources. For example, given there is sometimes a disconnect between staffing models used for hiring versus the perceptions of workload by physicists (e.g., type of clinical work), many of the qualitative responses from the focus groups and contextual inquiries noted that inadequate staffing often led to excessive workload as a result of not having enough medical physicists to cover the workload. This, in turn, impacted work‐life integration and time pressure, as the workload disrupted personal time and caused the feeling of not having enough time in the normal work‐day to complete their work. These associations suggest that some of the segregation of factor types into categories may be somewhat arbitrary and imprecise based on the validated affinity model.

Severity and priority of factors in our study did not always correspond, demonstrating that while a factor may be considered more severe, it was not necessarily a priority at that time to address. These results may have been influenced by medical physicists’ perceived readiness and motivation of leadership to address certain factors. For example, work‐life integration was considered a top factor in terms of severity but was ranked fourth potentially due to a perception that leadership may not be able to improve individual work‐life integration at that time. Medical physicist priorities and factors impacting well‐being in this current study are consistent with a prior reports where medical physicists were noted to have relatively high workloads, and prevalent factors included time demands, interruptions, and technical factors.^1^ This may suggest that many of these factors impacting well‐being are prevalent among medical physicists longitudinally, and that they are perhaps somewhat inherent to the job (potentially suggesting that improvement efforts may be challenging).

Factors were rated by level of perceived impact and effort by medical physicists at a total of four institutions, suggesting that the identified factors may be applicable to medical physicists at other US academic medical institutions, although sample size (*n* = 22) prevents broader generalizability. Medical physicists at the four institutions all identified that a hybrid model of work, improvements to scheduling workflows, and organizational culture were high impact, low effort, priorities to improve well‐being, and might help to focus feasible recommendations to organizational leadership across institutions that were not provided in previous studies assessing well‐being among medical physicists.^6^, ^9^ In an ideal scenario, each institution would use the systems‐analysis approach to “drill‐down” into the factors associated with well‐being and priorities for improvement at a local level. However, to protect participants in each institution, this study presents the results in aggregate per IRB directive.

## 5 Limitations

The study included four medical physics divisions at US academic medical centers and had a relatively small number of participants. This study was limited to US academic medical centers and does not capture factors related to well‐being among medical physicists in community hospitals and clinics. Nonetheless, the smaller number of participants involved in the initial survey, focus groups, contextual inquiries, and validation and prioritization sessions allowed us to comprehensively and contextually explore factors impacting well‐being among medical physicists in this study. Specifically, the diversity in methods used enabled us to exhaustively explore factors related to well‐being, supported by high percentages of agreement on the affinity model. Although expanding the participant pool to include other departmental roles could have increased the sample size, we elected not to do this since restricting the study to only medical physicists allowed for conclusions to be specific for medical physicists. We believe this is warranted since medical physicists have relatively unique job responsibilities.

Due to the small sample size, some of the qualitative comments from the survey, focus groups, and contextual inquiries were redacted/removed by the research team. This was done to maintain the privacy of participants as per the guidelines set by the IRB. This limited our analysis for this manuscript. Although participants were able to respond anonymously on the survey and provide their individual thoughts and feedback during the contextual inquiries and prioritization sessions, there was potential for bias due to possible information sharing between participants, potentially influencing responses. Despite these limitations, the overall study design was well received by medical physicist participants, who generally appreciated the diversity in data collection methods. Thus, we believe the findings of our study are still of significance because of the diverse data collection methods and successful validation of the data by medical physicists. Our study used a participatory, data‐driven, and systems‐focused approach based on contextual design that appears to be one of the first and largest of its kind in our field.

## 6 CONCLUSION

Prioritized factors that could improve the well‐being of medical physicists include work‐life integration, scheduling workflows, and relationships with hospital‐level leadership. These factors seem to be within the control of each radiation oncology center. Our approach to identifying factors contributing to well‐being aligns with the overall vision of maintaining the highest possible standards of patient safety in radiation therapy quality management^8^ in two important ways: (1) recognizing that medical physicist well‐being is crucial for promoting patient safety and quality of care, and (2) well‐being issues may inform future needs of quality management programs in radiation therapy. Future multi‐institutional research and replication with medical physicists at community hospitals or clinics would likely help establish the generalizability of our findings and support the development and assessment of systems‐focused interventions to enhance medical physicists well‐being. There is also a need to further define optimal hybrid models of work to support medical physicists well‐being.

## AUTHOR CONTRIBUTIONS

Karthik Adapa, Elizabeth Kwong, and Lukasz Mazur designed the study. Elizabeth Kwong and Chao Chin Liu performed data collection and analyzed the data. Elizabeth Kwong, Chao Chin Liu, and Lukasz Mazur interpreted the data. Elizabeth Kwong prepared the first draft of the manuscript. Lukasz Mazur provided overall supervision for conceptualization, analysis, methodology, and reviewing and editing of the manuscript. All authors (Elizabeth Kwong, Chao Chin Liu, Karthik Adapa, Lisa Vizer, Brian Anderson, Damian McHugh, Todd Pawlicki, Moyed Miften, Amit Sawant, Nadia Charguia, Shiva Das, Lawrence B. Marks, Jean L.Wright, and Lukasz Mazur) read and approved the final manuscript. Lukasz Mazur serves as the guarantor.

## CONFLICT OF INTEREST STATEMENT

The authors declare no conflicts of interest.

## CLINICAL TRIAL INFORMATION

None

## Supporting information



Supplementary Materials

## Data Availability

Additional research data are stored in an institutional repository and may be shared upon request to the corresponding author.
