# Supplementary material for: Towards better understanding of factors contributing to medical physicist well‐being in academic medical centers: A systems‐analysis approach
Source: J Appl Clin Med Phys. 2025 May 11;26(6):e70122. doi: 10.1002/acm2.70122 (PMC12148786; doi:10.1002/acm2.70122)
Supplement: Supplementary file 1 — Supplementary Materials [file ACM2-26-e70122-s001.pdf]

Towards Better Understanding of Factors Contributing to Medical Physicist Well-being in Academic Medical Centers: A Systems-Analysis Approach.

Supplementary Materials

INTRODUCTION AND CONSENT

Thank you for agreeing to participate in this survey. The purpose of this survey is to gather information and develop an understanding of the factors contributing to healthcare professionals. The results will be used to guide targeted improvement efforts aimed at improving well-being and reducing workplace burdens.

Your responses are confidential and will never be shared individually, and will only be reported in aggregate.

We appreciate you taking the time to provide your insights into the day-to-day life of a healthcare professional. There are a total of 5 pages, and should take you between 15 and 20 minutes to complete.

Note: We recommend taking this survey on a laptop / desktop for better view and ease of responding.

Please click YES if you agree to participate and NO if you do not. You can review the full CONSENT FORM HERE .

- ○ YES
- ○ NO

DEMOGRAPHICS

Part 1: Please answer the following questions related to demographics. We respect your privacy. If you don't want to share any specific information, please use the *"Prefer not to disclose"* option.

How many years have you been in your current position?

- ○ < 1
- ○ 1 - 2
- ○ 3 - 4
- ○ 5 - 9
- ○ 10 - 14
- ○ 15 - 20
- ○ > 20
- ○ Prefer not to disclose

Typically, how many hours per week do you work?

- ○ < 40
- ○ 40 - 49
- ○ 50 - 59
- ○ 60 - 69
- ○ 70 - 79
- ○ > = 80
- ○ Prefer not to disclose

Which age group do you belong to?

- ○ <25
- ○ 25-34

- ○ 35-44
- ○ 45-54
- ○ 55-65
- ○ >=65
- ○ Prefer not to disclose

What best describes your gender?
- ○ Male
- ○ Female
- ○ Non-binary/third gender
- ○ Transgender Male
- ○ Transgender Female
- ○ Prefer to self-describe:____________________
- ○ Prefer not to disclose

Which of the following best represents your racial or ethnic heritage? Select all that apply.
- ☐ White or Caucasian
- ☐ Black or African American
- ☐ Hispanic, Latino, Latina, or Latinx
- ☐ Asian
- ☐ American Indian or Alaska Native
- ☐ Native Hawaiian or other Pacific Islander
- ☐ Prefer to self-describe:______________________
- ☐ Prefer not to disclose

Which of the following best represents your marital status?
- ○ Single
- ○ Married
- ○ Divorced
- ○ Separated
- ○ Widowed
- ○ Prefer to self-describe:_______________________
- ○ Prefer not to disclose

Part 2: Please rate the following indicators of resilience and burnout.

I am able to adapt when changes occur.
- ○ Not true at all
- ○ Rarely true
- ○ Sometimes true
- ○ Often true
- ○ Always true

I tend to bounce back after illness, injury, or other hardships.
- ○ Not true at all
- ○ Rarely true
- ○ Sometimes true
- ○ Often true
- ○ Always true

I feel burned out from my work.
- ○ Not true at all
- ○ Rarely true
- ○ Sometimes true
- ○ Often true
- ○ Always true

I have become more callous toward people since I took this job. *(Rate how often you generally feel this way.)*
- ○ A few times a year or less
- ○ Once a month or less
- ○ A few times a month
- ○ Once a week
- ○ A few time a week
- ○ Every day

WORKPLACE FACTORS CURRENT STATUS

Please read the instructions before beginning this section.

Part 3: Please answer the following questions related to the factors that might be contributing to your workplace stress

This page lists workplace factors that contribute to provider burnout and well-being (each row corresponds to a factor, listed in **bold** … factor, representative examples have been provided for context.

Your responses and comments on these factors will help us determine workplace improvement activities and guide follow-up focus groups.

INSTRUCTIONS

For each factor, please provide your responses in the following columns:
1. **Current Status**: Please rate the degree to which these workplace factors have contributed to your stress over the last 3 months.
2. **Specific Examples**: Please use this space to indicate the specific issues / frustrations you experience related to this factor, especially when you rate *High* or *Extremely High*.

	**Current Status** (Rate the degree to which these factors contribute to your workplace stress)					**Specific Examples** Please use this space to indicate the specific issues / frustrations you experience related to this factor, especially when you rated *High* or *Extremely High*.
	Not at all	Low	Moderate	High	Extremely High	
**Excessive workload** Amount of work to be performed exceeds the time in which to do the work; chronic high level of mental workload (divided attention, feeling rushed, information and communication overload); covering for infected colleagues.	○	○	○	○	○	
**Unmanageable work schedules** Excessive number of work hours on service, per week; Excessively long and continuous shifts (with post-shift overtime); amount of time / number of nights on-call per week; sleep deprivation	○	○	○	○	○	
**Time pressure** Constant, ever-present time pressure, feeling of chasing the clock; pressure to shorten time with patients; not enough time to complete clinical documentation during the workday.	○	○	○	○	○	
**Inadequate staffing**	○	○	○	○	○	

Perception of "short" staffing (too few support staff, too few providers / clinicians on service); chronic pressure to increase patient load.						
**Inefficient workflows**<br>Inefficient, poorly defined, or variable workflows (ambiguous Standard Operating Procedures (SOP), variations between departments / units); inefficiently implemented workflows in Epic (e.g., med rec, medication orders); cancelled, missing, or delayed labs; documenting and tracking down others' work (missing consults, recommendations, note bloat).	○	○	○	○	○	
**Interruptions & distractions**<br>Excessive interruptions (paging for non-emergent issues); inappropriate default values in Epic; navigating changes due to Epic updates causes interruptions; patient portal messages and lab results; patient family questions outside the engagement time; excessive, poorly timed electronically generated messages; alerts and notifications; Vocera (wrong calls, loud volume)	○	○	○	○	○	
**Inadequate technology implementation**<br>Epic; Vocera; pages; direct instant messaging; lack of integration between various applications within and outside Epic; lack of at-the-elbow IT support	○	○	○	○	○	
**Moral distress**<br>Extending life beyond patient wishes / family pressure – treatment at all costs; rationing healthcare – who gets treated; high volume of COVID-related deaths; restriction of visitors; dying alone.	○	○	○	○	○	
**Patient factors**<br>Verbally abusive patients / family; physically abusive patients / family; High-acuity patients require more clinical and non-clinical work.	○	○	○	○	○	
**Lack of recognition for Quality Improvement activities**<br>Lack of dedicated time; lack of recognition for engagement in Quality Improvement activities.	○	○	○	○	○	
**Lack of dedicated time for Professional Development requirements**<br>Lack of dedicated time for "necessary" professional development activities such as CME, re-certification, and Training: HIPAA, infection control, patient safety, etc.	○	○	○	○	○	
**Administrative burden**<br>Excessive amount of time spent on administrative tasks (clinical (e.g., looking up labs) and non-clinical (e.g., billing)); administrative burden takes time away from meaningful clinical care (patient interaction).	○	○	○	○	○	
**Lack of support for Research and Teaching**<br>Lack of protected time for research and teaching activities; lack of recognition for research activities and teaching activities.	○	○	○	○	○	
**Professional relationships**<br>Poor interpersonal relationships with outside group colleagues / across disciplines; co-worker incivility, bullying, or abuse; lack of inter-relational coordination (communicating and relating to complete a task); lack of social support from administration; lack of collegial social support (peers); emotional contagion (feeling better / worse after interaction with positive / angry individual).	○	○	○	○	○	
**Organizational culture**<br>Low leadership / management support; lack of leadership in setting tone for interprofessional conduct and collaboration; lack of fairness / equity in organizational policies and procedures.	○	○	○	○	○	
**Physical work environment**<br>Unsatisfactory physical work environment; insufficient workspace; inadequate workstations / equipment; lack of adequate meeting space.	○	○	○	○	○	
**Values and expectations alignment**<br>Inconsistent / unaligned values (unrealistic productivity measures vs. delivering high-quality excellent care); time spent on tasks / activities below license.	○	○	○	○	○	
**Job control (flexibility and autonomy)**	○	○	○	○	○	

Low sense of control over one's practice environment; little autonomy over how one performs one's work; lack of decision-making authority (e.g., in care delivery); inflexible work schedule; lack of involvement in shared governance (not having an influence on institutional policy).						
**Intrinsic motivations and rewards**<br>Threats to intrinsic motivations such as meaning, purpose and joy in work, intellectual stimulation, engagement, sense of helping / healing.	○	○	○	○	○	
**Extrinsic motivations and rewards**<br>Threats to extrinsic motivations such as money, prestige, praise; lack of feeling appreciated (by supervisors, peers, patients); lack of feeling respected (by administration, peers, patients).	○	○	○	○	○	
**Work-life integration**<br>Struggles with work-life integration; work-home conflict; childcare concerns; schedules do not allow sufficient time away from office; necessity to use Epic remotely from home after work hours (pajama time); on-call schedule disrupts dedicated personal time.	○	○	○	○	○	

WORKPLACE FACTORS PRIORITIZATION

Please read the instructions before beginning this section

PART 3 (continued): Please prioritize the workplace factors as they apply to you.

This page lists the same workplace factors you saw on the previous screen.

INSTRUCTIONS

For each factor, please provide your responses in the following columns:

1. **Priority for Improvement**: Rate the priority you believe the department should take in addressing and improving this factor. The higher you rate the priority, the greater urgency / need for targeting this factor for improvement.
2. **Comments**: Please use this space to express and thoughts related to the prioritization of these factors, especially when you rate *High Priority*.

	**Priority for Improvement** (More urgent factors should receive higher priority ratings)				**Comments** (Please provide reasons for your rating)
	Not an issue	Low Priority	Medium Priority	High Priority	
**Excessive workload**<br>Amount of work to be performed exceeds the time in which to do the work; chronic high level of mental workload (divided attention, feeling rushed, information and communication overload); covering for infected colleagues.	○	○	○	○	
**Unmanageable work schedules**<br>Excessive number of work hours on service, per week; Excessively long and continuous shifts (with post-shift overtime); amount of time / number of nights on-call per week; sleep deprivation	○	○	○	○	
**Time pressure**<br>Constant, ever-present time pressure, feeling of chasing the clock; pressure to shorten time with patients; not enough time to complete clinical documentation during the workday.	○	○	○	○	
**Inadequate staffing**<br>Perception of "short" staffing (too few support staff, too few providers / clinicians on service); chronic pressure to increase patient load.	○	○	○	○	
**Inefficient workflows**	○	○	○	○	

Inefficient, poorly defined, or variable workflows (ambiguous Standard Operating Procedures (SOP), variations between departments / units); inefficiently implemented workflows in Epic (e.g., med rec, medication orders); cancelled, missing, or delayed labs; documenting and tracking down others' work (missing consults, recommendations, note bloat).					
**Interruptions & distractions**<br>Excessive interruptions (paging for non-emergent issues); inappropriate default values in Epic; navigating changes due to Epic updates causes interruptions; patient portal messages and lab results; patient family questions outside the engagement time; excessive, poorly timed electronically generated messages; alerts and notifications; Vocera (wrong calls, loud volume)	○	○	○	○	
**Inadequate technology implementation**<br>Epic; Vocera; pages; direct instant messaging; lack of integration between various applications within and outside Epic; lack of at-the-elbow IT support	○	○	○	○	
**Moral distress**<br>Extending life beyond patient wishes / family pressure – treatment at all costs; rationing healthcare – who gets treated; high volume of COVID-related deaths; restriction of visitors; dying alone.	○	○	○	○	
**Patient factors**<br>Verbally abusive patients / family; physically abusive patients / family; High-acuity patients require more clinical and non-clinical work.	○	○	○	○	
**Lack of recognition for Quality Improvement activities**<br>Lack of dedicated time; lack of recognition for engagement in Quality Improvement activities.	○	○	○	○	
**Lack of dedicated time for Professional Development requirements**<br>Lack of dedicated time for "necessary" professional development activities such as CME, re-certification, and Training: HIPAA, infection control, patient safety, etc.	○	○	○	○	
**Administrative burden**<br>Excessive amount of time spent on administrative tasks (clinical (e.g., looking up labs) and non-clinical (e.g., billing)); administrative burden takes time away from meaningful clinical care (patient interaction).	○	○	○	○	
**Lack of support for Research and Teaching**<br>Lack of protected time for research and teaching activities; lack of recognition for research activities and teaching activities.	○	○	○	○	
**Professional relationships**<br>Poor interpersonal relationships with outside group colleagues / across disciplines; co-worker incivility, bullying, or abuse; lack of inter-relational coordination (communicating and relating to complete a task); lack of social support from administration; lack of collegial social support (peers); emotional contagion (feeling better / worse after interaction with positive / angry individual).	○	○	○	○	
**Organizational culture**<br>Low leadership / management support; lack of leadership in setting tone for interprofessional conduct and collaboration; lack of fairness / equity in organizational policies and procedures.	○	○	○	○	
**Physical work environment**<br>Unsatisfactory physical work environment; insufficient workspace; inadequate workstations / equipment; lack of adequate meeting space.	○	○	○	○	
**Values and expectations alignment**<br>Inconsistent / unaligned values (unrealistic productivity measures vs. delivering high-quality excellent care); time spent on tasks / activities below license.	○	○	○	○	
**Job control (flexibility and autonomy)**<br>Low sense of control over one's practice environment; little autonomy over how one performs one's work; lack of decision-making authority (e.g., in care delivery); inflexible work schedule; lack of involvement in shared governance (not having an influence on institutional policy).	○	○	○	○	

**Intrinsic motivations and rewards**<br>Threats to intrinsic motivations such as meaning, purpose and joy in work, intellectual stimulation, engagement, sense of helping / healing.	○	○	○	○	
**Extrinsic motivations and rewards**<br>Threats to extrinsic motivations such as money, prestige, praise; lack of feeling appreciated (by supervisors, peers, patients); lack of feeling respected (by administration, peers, patients).	○	○	○	○	
**Work-life integration**<br>Struggles with work-life integration; work-home conflict; childcare concerns; schedules do not allow sufficient time away from office; necessity to use Epic remotely from home after work hours (pajama time); on-call schedule disrupts dedicated personal time.	○	○	○	○	

ADDITIONAL WORKPLACE STRESSORS

Please read the instructions before beginning this section

PART 4: Please share any ADDITIONAL FACTORS which you feel were not covered in the previous section but are important and need to be highlighted.

For any additional factors that you mention in this section, please provide your responses (ratings and priority), as you did for the previous factors.

List in the spaces below any other factors specific to your work that you find frustrating, broken, annoying, or burdensome.	**Current Status** (Rate the degree to which these factors contribute to your workplace stress)					**Priority for Improvement** (More urgent factors should receive higher priority ratings)			
	Not at all	Low	Moderate	High	Extremely High	Not an issue	Low Priority	Medium Priority	High Priority
	○	○	○	○	○	○	○	○	○
	○	○	○	○	○	○	○	○	○
	○	○	○	○	○	○	○	○	○
	○	○	○	○	○	○	○	○	○
	○	○	○	○	○	○	○	○	○

ADDITIONAL COMMENTS

Please use this space to share any other thoughts, feedback, or concerns.
(This is the last question. Please do not click “Proceed to next screen” if you haven’t completed the survey.)

Figure A. Electronic survey administered to medical physicists.

[Virtual] Focus Group Guide
Radiation Oncology – Medical Physics

** Ensure Focus Group slides are pulled up on the screen before starting **

Hi all,

Thank you for joining us today for our Wellbeing Deep Dive Focus Group. My name is [NAME] and I will be moderating our focus group session today. If at any point during our hour together you need assistance with anything or have a question, please don't hesitate to ask.

With your consent, we will be recording today's focus group. The recorded portion will be for research purposes only and will not be shared outside of myself and the research team. Do you consent to being recorded? ** Wait for response ** Thank you.

** Start recording; go to slide 2, Agenda **

Agenda
The purpose of this focus group is to get your thoughts, insights, and contextual information on system-related factors contributing to physicists' burnout. We don't want you to solve the problems. Rather, we would like you to provide more details on what factors are contributing to your burnout.

During our one hour today, we will be:
- Viewing the quantitative data from the survey to identify top workplace stressors;
- Reviewing text responses on top stressors and providing contextual information and details; and
- Ranking which workplace stressors are your highest priority to address.

I will be summarizing the survey results based on the individual Qualtrics survey you filled out, and outlining what factors were rated as the most severe. These will include qualitative data in the form of text responses based on the top rated factors on the surveys. As mentioned on the email with the survey link, text responses are anonymous.

** Move to Survey Results slides **

Survey Results
The survey was sent to [#] physicists at [institution], with [#] completing the survey as of Tuesday for a completion rate of [%].

** Move to Workplace Stressors slides **

Workplace Stressors: Rating and Priority Ranks
Workplace stressors were rated in terms of severity and priority. This slide shows the aggregated data from the survey results and the top workplace stressors in terms of severity, and by priority. We will specifically be looking at data today that have the highest averages in terms of severity.

The next slide gives a closer look at the top workplace stressors by severity, with Inadequate Staffing and Work-life Integration tied as the top stressors with scores of 3.13 on a 5-point Likert scale.

[What are your thoughts on these results? Do you agree with them? Are you surprised by them?]

The next slide provides more detailed data on those top stressors by severity, with averages, range, and standard deviations provided. Next, we will be looking at text responses from the survey for these top stressors. There will not be any identifying information on the slides.

** Move to quotes slides **

Workplace Stressors: Text Responses

For each workplace factor's text responses, we would like you to read through them on the screen and provide any contextual information or specific details to elaborate on the responses. This will allow us to get a more holistic and comprehensive view of the top workplace stressors.

The first top workplace stressor we will be starting with is Inadequate Staffing. Please take the next couple minutes or so to read through some of the quotes, and let me know your thoughts on the information on the slide.

** Pause while they read. Wait for responses; if the conversation isn't going, ask pointed questions below: **

What are your thoughts on the quotes? Do you agree with them? Disagree with them?
What about this specific quote here? Could you tell me more about that?

** Once sufficient time has been spent on the slide [~5-8 minutes], move to next slide **

The next top workplace stressor we will be looking at quotes for is [Work-life Integration]. Please take the next couple minutes or so to read through some of the quotes, and let me know your thoughts on the information on the slide.

** Repeat questions & process above until you get through all the text responses, OR you only have 5-10 min left **
** Move to Priority Scores slide **

Prioritizations

Thank you for providing your thoughtful and honest responses to the text responses. Now that we have reviewed the qualitative data from the top workplace stressors by severity, we will next look at prioritizations on what you feel should be prioritized and addressed for improvement. These Priority Scores from the survey show Inadequate Staffing was rated as the top workplace stressor to be addressed.

[What are your thoughts on these results? Do you agree with them? Are you surprised by them?]

The next slide has a more detailed look into the top workplace stressors with the highest priorities by average, with range and standard deviation also provided.

Prioritization Poll

** Put Qualtrics poll link in chat before reading. **

Finally, the last portion of our focus group today will be a brief, one-question Prioritization poll. I just put a quick Qualtrics link in the chat. Now that we have taken the time to discuss top workplace stressors, please take the next 1 minute or so to rank them by your individual priority to be addressed. This will help us throughout our process and will help focus future recommendations that will be provided to system leadership.

Wrap Up

Thank you for taking the time to participate in our well-being deep dive focus group.

If you have not already completed it yet, please make sure to complete the Qualtrics Well-being survey, which will help inform on individual thoughts and priorities regarding the stressors we talked about today and more.

Additionally, we will be conducting shadowing sessions starting next week for the next few weeks for you all to share more individual thoughts and opinions. This provides an opportunity for our team to shadow you during your day-to-day work activities at a time that is convenient for you, and allows us to understand breakdowns and workarounds in your respective areas. We will be working with the division coordinator to set up shadowing times that might fit your schedule, and I will reach out via email or text to coordinate a date and time that works for you.

That concludes our well-being deep dive focus group. I will stick around if you have any questions. Otherwise, please have a great rest of your day!

** End recording **

Figure B. Focus group guide.

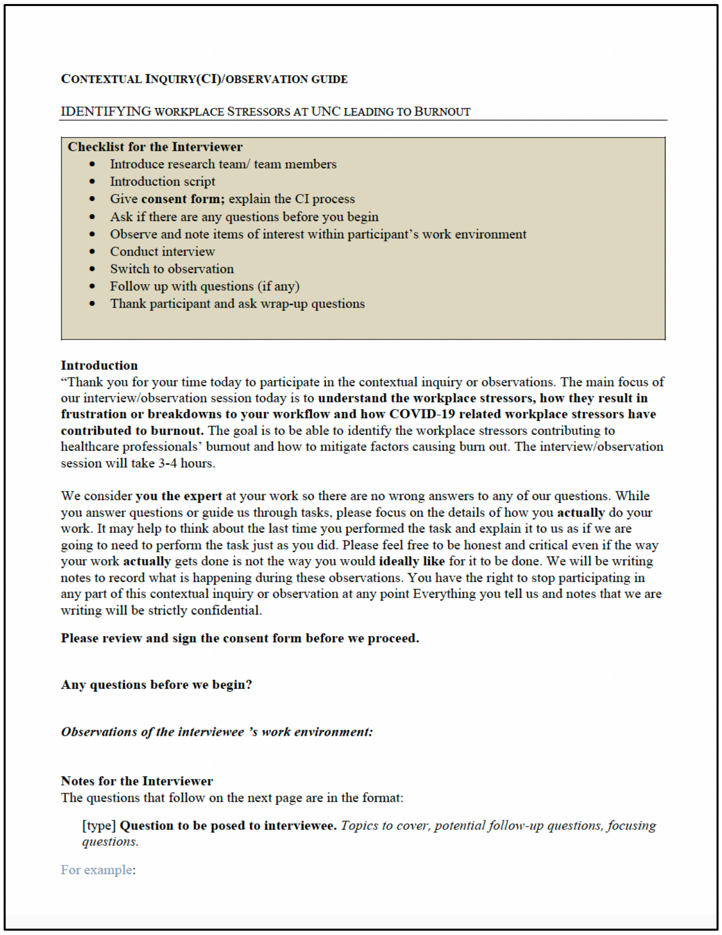

CONTEXTUAL INQUIRY(CI)/OBSERVATION GUIDE

IDENTIFYING WORKPLACE STRESSORS AT UNC LEADING TO BURNOUT

Checklist for the Interviewer

- Introduce research team/ team members
- Introduction script
- Give **consent form;** explain the CI process
- Ask if there are any questions before you begin
- Observe and note items of interest within participant's work environment
- Conduct interview
- Switch to observation
- Follow up with questions (if any)
- Thank participant and ask wrap-up questions

Introduction

"Thank you for your time today to participate in the contextual inquiry or observations. The main focus of our interview/observation session today is to **understand the workplace stressors, how they result in frustration or breakdowns to your workflow and how COVID-19 related workplace stressors have contributed to burnout.** The goal is to be able to identify the workplace stressors contributing to healthcare professionals' burnout and how to mitigate factors causing burn out. The interview/observation session will take 3-4 hours.

We consider **you the expert** at your work so there are no wrong answers to any of our questions. While you answer questions or guide us through tasks, please focus on the details of how you **actually** do your work. It may help to think about the last time you performed the task and explain it to us as if we are going to need to perform the task just as you did. Please feel free to be honest and critical even if the way your work **actually** gets done is not the way you would **ideally like** for it to be done. We will be writing notes to record what is happening during these observations. You have the right to stop participating in any part of this contextual inquiry or observation at any point Everything you tell us and notes that we are writing will be strictly confidential.

Please review and sign the consent form before we proceed.

Any questions before we begin?

Observations of the interviewee 's work environment:

Notes for the Interviewer

The questions that follow on the next page are in the format:

[type] **Question to be posed to interviewee.** *Topics to cover, potential follow-up questions, focusing questions.*

For example:

[Motivation] **What do you enjoy most about your job as [role]?** *Why? What activities do you always tackle first?*

Types of questions:

[Variables] – Verifying demographic and behavioral variables

[Mental Model] – How does the participant think about work, resources, services & systems? What are the key barriers and facilitators at workplace?

[Activities] – What kinds of activities does the participant perform? Do they occur regularly or occasionally?

[Motivation] – What are the interviewee's goals? What does the interviewee enjoy doing?

[Opportunities] – What are the interviewee's pain points? What do they avoid?

[Interactions] – Who or what does the participant consult or work with?

[Process] – How does the participant make decisions and perform tasks?

Interview Portion

Warm-up Questions

[Variables] **How long have you been at** *[participant 's institution]***?**

[Variables] **How long have you been working at the location?**

[Variables] **What is your primarily role?**

[Variables] **What are the different healthcare professionals with whom you work closely?**

[Variables] **How do you communicate with other co-workers, what are the technology/tools that you use to communicate with other care team members and with patients***]? How long have you been using it? Have you used any others?*

[Opportunities]**What are the key workplace stressors? Why?**

General Questions

[Activities] **What do you do as healthcare professional? Could you please describe a typical day for you at work?** *Which activities occur regularly? Which activities occur only on occasion?*

[Motivation] **What do you enjoy most about your job as a healthcare professional?** *Why? What activities do you always tackle first?*

[Opportunities] **What do you enjoy least about your job as a healthcare professional?** *Why? What activities currently waste your time? What are the factors at your workplace that impede your performance?*

[Activities] **Can you tell us more about key stressors at your workplace? Can you help us understand what are the other factors that contribute to work place stress**

[Variables] **How many colleagues work with you closely? What are the different roles that you play as a care team**

[Variables] **How large is the care team? How often does it meet?**

[Interactions, Process] **How do you work with your colleagues? What are the modes of communication with other team members?** *What do you and your colleagues and co-workers do together?*

[Activities, Mental Model] **We're interested in understanding physical, social, cultural and psychological factors or activities or tasks contributing to burnout. Which of these types of activities do you actively participate? How frequently do these activities take place?**

[Goals] **What is/are the goal(s) behind these activities or tasks or factors?**

[Opportunities] **Any frustrations with your current process of creating, deploying, collecting or evaluating these activities? What frustrations do you have with this within?**

Observation Portion

In this part, the research team acts as apprentices observing as the participant walks through an activity or tasks on EHR or any other technology or tool, interrupting with questions only occasionally. The goal is for the research team to be able to re-create the work of the participant. **The activity of interest should be one that the interviewee has said contribute to burn out and result in frustration to the participant or breakdown of the workflow.**

For example, the participant may find a specific module or functionality with electronic health records (EHR) as extremely frustration. Given this example, the research may request the participant to provide a running description of how the HER interface causes distress or frustration or breakdown of the workflow, how they try to reach out to colleagues and other resources to overcome the problem related to workflow and how that results in delayed care to patient.

Participants will be informed that research team would like to observe them giving us a running description of how that specific module or functionality within EHR causes frustration to participant. extremely frustration. They will be requested to please walk through this activity just as they performed it last, thinking aloud and letting us know why they are doing what you are doing. Participants will asked to help research team understand how they do their work in EHR so that they may re-create it.

Participant will be requested to focus on the activity from beginning to end, with emphasis on thinking aloud. If time does not allow for the completion of the activity, participants will be asked how they would finish the activity.

The research team shall avoid interrupt the participants and will request for some additional only under rare circumstances such as :

- The participant is doing a lot of work without speaking about it; participant will be reminded to give a running description of what they are doing so that research team, may be able to re-create their work.
- The interviewee is giving very general or broad details about an important task or action; participant will be reminded to recount the details of the last time they went through this activity to make it more concrete.

Important areas that research team would want to see/hear described

Creating Activity

- Do you collaborate with others to create the activity? In what way?
- Do you re-use the activity? When? How (often)?

Mistakes in performing the tasks/activity (*omit if not applicable to role*)

- What are the common mistakes that healthcare professional makes and how do they recover ?

Completing Activity (*omit if not applicable to role*)

- What are the workarounds for task(s) that are either not well supported by system in use or not available within system in use and therefore user has to rely on another system to complete task(s)

Do you share these activities with others?

- With whom?
- How?
- Within department/program?

Observation Notes:

Areas to focus on:

- **Sequences** – in what order is the participant performing tasks? Are there interruptions? Are they doing more than one thing at a time?
- **Tools** – what is the participant using to perform their work?
- **Problems** – what is frustrating or unexpected for the participant?
- **Mental models** – how does the participant think about their work? What expectations do they have?

Interactions – do healthcare professionals consult different people, resources? If so, what for?

Follow-up Questions

In this part of the interview, the interview team takes a moment to reflect on the observation:

Are there gaps in the process the participants have described? The research team takes the time to investigate and make sure all the steps involved in completing the activity are recorded. The research team may end up delving back into observation mode as the interviewee remembers something they forgot to mention and begins another description of tasks and actions.

Are there other parts of the process that are unclear? The research team uses this time to clarify.

Were there instances where the interviewer or note-taker had a question, but did not ask because it would have adversely interrupted the interviewee? The research team uses this time to ask these questions.

Wrap-up Questions

What did you think of this interview? *Anything you did not understand? Anything that made you anxious?*

Is there anything else regarding the key workplace stressors contributing to healthcare burnout that you would like to add?

Would you be willing to be contacted with follow-up questions?

Thanks so much for your input. We really value your feedback

Figure C. Contextual inquiry guide.

Radiation Oncology Physics Well-being Validation Survey

Thank you for agreeing to participate in this survey.

The purpose of this survey is to validate previously generated priorities for improvement (improvement ideas) for efforts aimed at improving well-being and reducing workplace burdens and frustrations. The results will be used to guide targeted improvement efforts.

Your responses are confidential and will never be shared individually, and will only be reported in aggregate.

We appreciate you taking the time to provide your insights and priorities. This brief validation survey should take you approximately 5 minutes to complete.

Note: We recommend taking this survey on a laptop / desktop for better view and ease of responding.

Please click YES if you agree to participate and NO if you do not. By clicking "YES" and "Continue" you consent to participate in the survey and study. You can review the full CONSENT FORM HERE .

○ YES ○ NO

The following priorities below are pre-generated priorities (improvement ideas) and their descriptions. The purpose of this brief survey is to understand **you and your organization**'s perceptions of these priorities.

We would like you to organize these priorities against two variables:

- **Level of Impact**- how much value or impact the outcomes will have on the Division of Medical Physics
- **Level of Effort** - how much time, money, resources, and capacity will be needed to achieve the desired outcome

Please rank priorities as lowest impact or effort, medium impact or effort, or highest impact or effort.

For the **empty boxes below**: Please share any **ADDITIONAL IMPROVEMENT IDEAS** which you feel were not covered but are important and need to be highlighted. For any additional improvement ideas that you add, please also rank them (low, medium, or high) for impact and effort.

Thank you for your time and thoughtful assessment of these improvement ideas! If you have any questions, please reach out to [research team] at [email].

What institution do you currently work at?

- ○ [redacted]
- ○ [redacted]
- ○ [redacted]
- ○ [redacted]
- ○ Other: _________
- ○ Prefer not to disclose

For each of the following faculty improvement ideas, please select the appropriate **Level of Impact** (how much value or impact the outcomes will have on your division) and **Level of Effort** (how much time, money, resources, and capacity will be needed to achieve the desired outcome) if implemented.

	**Level of Impact** (how much value or impact the outcomes will have on the division)			**Level of Effort** (how much time, money, resources, and capacity will be needed to achieve the desired outcome)		
	**Low Impact-** fewer coworkers impacted, shorter-term impact, affects one group or metric	**Medium Impact**	**High Impact-** more long-term gain, more coworkers impacted, more metrics affected	**Low Effort-** fewer coworkers needed to implement, less cost/time/resource intensive	**Medium Effort**	**High Effort-** more cost, time or resource intensive, more system involvement (ISD, HR, other) required, more co-workers needed to implement
**1. Have/maintain a hybrid model of work -** Working from home a few days a week would be / is beneficial to my work-life balance.	○	○	○	○	○	○
**2. Inadequate Staffing -** More physicists to cover clinical responsibilities and ease workload.	○	○	○	○	○	○
**3. Limit excessive workload -** Work that continues into the evening due to a tight treatment schedule impacts work-life balance.	○	○	○	○	○	○
**4. Improve scheduling workflow -** Ensure schedule is out 1 month in advance and more quickly.	○	○	○	○	○	○
**5. Improve organizational culture** from hospital leadership and admin (e.g., improve communication and visibility).	○	○	○	○	○	○
**6. Extrinsic motivation** - Better pay/benefits, bonuses for extra work or time.	○	○	○	○	○	○

**7. Inadequate Technology -** Upgrade machines; machines should be more reliable.	○	○	○	○	○	○
8. Have a **local / on-site / dedicated engineer** to address machine issues more quickly.	○	○	○	○	○	○
**9. Increase protected / dedicated time -** For training, educational responsibilities, teaching students, time dedicated towards policies and procedures, time allocation for getting tasks done.	○	○	○	○	○	○
**10. Inadequate Technology Integration -** Ensure people logging issues follow up with engineers to get the issues fixed.	○	○	○	○	○	○
**11. Improve meeting efficiency -** Meetings should be more efficient and a meaningful use of time.	○	○	○	○	○	○
**LIST YOUR OWN -** If applicable, provide any additional factors or improvement ideas specific to your work and categorize their level of Impact and Effort.	○	○	○	○	○	○
(List your own, if applicable)	○	○	○	○	○	○
(List your own, if applicable)	○	○	○	○	○	○

Figure D. Impact/Effort rating survey.
